# Variation in endogenous oxidative stress in *Escherichia coli* natural isolates during growth in urine

**DOI:** 10.1186/1471-2180-12-120

**Published:** 2012-06-22

**Authors:** Cecile Aubron, Jeremy Glodt, Corine Matar, Olivier Huet, Didier Borderie, Ulrich Dobrindt, Jacques Duranteau, Erick Denamur, Marc Conti, Odile Bouvet

**Affiliations:** 1 UMR 722 INSERM and Université Paris Diderot, Sorbonne Paris Cité, Faculté de Médecine, Site Xavier Bichat, Paris, France; 2 Laboratoire de Biochimie A, Assistance Publique - Hôpitaux de Paris and Université Paris Sud, Hôpital Bicêtre, Le Kremlin-Bicêtre, France; 3 Département d’Anesthésie Réanimation, Assistance Publique - Hôpitaux de Paris and Université Paris Sud, Hôpital Bicêtre, Le Kremlin-Bicêtre, France; 4 Laboratoire de Biochimie, Assistance Publique - Hôpitaux de Paris and Université Paris Descartes, Hôpital Cochin, Paris, France; 5 Institute for Hygiene, University of Münster, Münster, Germany; 6 Bioquanta, Mitoxis, France; 7 Université Paris-Est, INSERM U955 EQ07, Créteil, France

**Keywords:** *Escherichia coli*, Urine, Oxidative stress, Adaptation, Diversity

## Abstract

**Background:**

Uropathogenic strains of *Escherichia coli* cause symptomatic infections whereas asymptomatic bacteriuria (ABU) strains are well adapted for growth in the human urinary tract, where they establish long-term bacteriuria. Human urine is a very complex growth medium that could be perceived by certain bacteria as a stressful environment. To investigate a possible imbalance between endogenous oxidative response and antioxidant mechanisms, lipid oxidative damage estimated as thiobarbituric acid reactive substances (TBARS) content was evaluated in twenty-one *E. coli* belonging to various pathovars and phylogenetic groups. Antioxidant defense mechanisms were also analysed.

**Results:**

During exponential growth in urine, TBARS level differs between strains, without correlation with the ability to grow in urine which was similarly limited for commensal, ABU and uropathogenic strains. In addition, no correlation between TBARS level and the phylogroup or pathogenic group is apparent. The growth of ABU strain 83972 was associated with a high level of TBARS and more active antioxidant defenses that reduce the imbalance.

**Conclusions:**

Our results indicate that growth capacity in urine is not a property of ABU strains. However, *E. coli* isolates respond very differently to this stressful environment. In strain ABU 83972, on one hand, the increased level of endogenous reactive oxygen species may be responsible for adaptive mutations. On the other hand, a more active antioxidant defense system could increase the capacity to colonize the bladder.

## Background

*Escherichia coli* is a highly versatile bacterial species. Commensal *E. coli* strains are normal inhabitants of the human colon [1], but pathogenic strains of *E. coli* can cause intestinal and extraintestinal diseases of which urinary tract infections (UTIs) rank first [2]. Population genetic studies based on both multi-locus enzyme electrophoresis and various DNA markers have identified four major phylogenetic groups A, B1, B2, and D and a potential fifth group E, among *E. coli* strains [3-5]. Several studies have demonstrated a relationship between pathogenicity and phylogenetic groups. Clones responsible for human extraintestinal infections frequently belong to B2, and to a lesser extent D, phylogenetic groups, whereas commensal population strains are most common in groups A and B1[6,7].

UTIs are the most common human infectious diseases and are a major cause of morbidity. It is estimated that there are about 150 million cases in the world per year [8]. Uropathogenic strains of *E. coli* (UPEC) are responsible for more than 80% of all UTIs [9]. Virulence factors, such as adhesins, toxins and siderophores enhance the ability of UPEC to cause UTIs [10]. The ability to grow in human urine is certainly also a necessary criterion for the colonization of the bladder [11]. Indeed, the ability of *E. coli* strains to survive and use resources available in urine efficiently is an important adaptation to the urinary tract [12]. This is illustrated by the asymptomatic bacteriuria (ABU) strains that colonise the urinary tract but do not cause disease. *E. coli* 83972, the ABU strain prototype, which is unable to express functional type 1, P and F1C fimbriae, grows extremely well in urine. Its growth rate is high enough to overcome the losses due to micturition [11].

**Figure 1:**
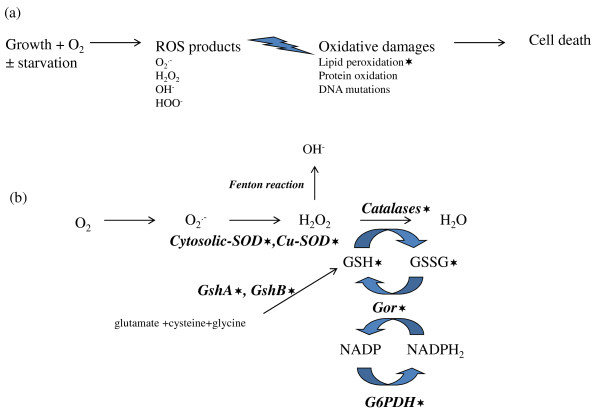
**Scheme illustrating the effects of reactive oxygen species (ROS) into the cell (a) and antioxidant defense mechanisms (b) evaluated in this work.** (**a**) Growth in the presence of reactive oxygen species (ROS): superoxide radical [O_2_^.-^], hydrogen peroxide [H_2_O_2_], hydroxyl radical [OH^-^] and hydroperoxyl radical [HOO^-^]. These ROS can damage nucleic acids (RNA and DNA) as well as proteins and lipids, leading to cell death. (**b**) The superoxide dismutases (SOD), which are cytosolic (Mn-SOD and Fe-SOD) and periplasmic (Cu-SOD) allow O_2_^.-^ detoxification. The catalase activity responsible for the reduction of H_2_O_2_ to H_2_O is effected by two hydroperoxydases [hydroperoxydase I (HPI) and hydroperoxydase II (HPII)], and the alkyl hydroperoxydase (AhpC). The glutathione is synthetised from glutamate, cysteine and glycine, by 2 unrelated ligases: the γ-glutamylcysteine synthetase (GshA) and the glutathione synthetase (GshB). The glutathione oxidoreductase (Gor) reduces glutathione disulfide (GSSG), which is formed upon oxidation. The glucose 6-phosphate dehydrogenase (G6PDH) allows indirectly the reduction of NADP to NADPH. The Fenton reaction is the result of electron transfer from donor to H_2_O_2_ catalyzed by iron Fe^3+^. The stars show dosages effected in this study.

Endogenous reactive oxygen species (ROS), such as hydrogen peroxide, superoxide anion radical and hydroxyl radicals are generated continuously in cells grown aerobically. They are responsible for damages on nucleic acids (RNA and DNA), as well as proteins and lipids, leading to cell death [13,14] (Figure 1a). Defense mechanisms against the damaging effects of oxidative stress involve both enzymatic components, such as catalases and superoxide dismutases (SOD), and nonenzymatic components, such as glutathione-dependant reduction systems (Figure 1b).

It has been demonstrated that growth under starvation conditions generates oxidative stress and significant changes in glutathione homeostasis [15-17]. The increased level of ROS comes from the imbalance between production and antioxidant mechanisms. Human urine is a high-osmolarity, moderately oxygenated, iron and nutrient-limited environment [18-21]. Therefore, growth in urine could be perceived as a stressful environment. In order to evaluate the importance of endogenous oxidative stress of growing cells in urine, oxidative damages to lipids were assayed in a range of *E. coli* strains representative of various pathovars and phylogenetic groups. Antioxidant defense mechanisms in four representatives of these *E. coli* strains were also analysed.

## Methods

### Strains

The twenty-one *E. coli* strains used in this study are described in Table 1; nine were pathogenic, three were commensal and nine ABU. The archetypal UPEC strain CFT073 was originally isolated from the blood and urine of a woman admitted to the University of Maryland Medical System [22]. Seven other UPEC prototypes, *E. coli* UTI 89, J96, UMN026, IAI39, IAI74, IH11128 and 536, were also studied [23-25]. *E. coli* ABU 83972, the archetypal ABU strain, is a clinical isolate capable of long-term bladder colonization [26]. Eight other ABU strains were also included [27]. *E. coli* K-12 MG 1655, a commensal, laboratory derived strain, was originally isolated from the faeces of a convalescent diphtheria patient in Palo Alto in 1922 [28]. *E. coli* IAI1 is a commensal strain isolated from faeces of a young, healthy, military conscript in the 1980s in France [6]. *E. coli* ED1a, isolated in the 2000s from faeces of a healthy man in France, belongs to a human-specific, widespread, commensal clone that is increasing in frequency [29]. Finally, an enterohaemorrhagic *E. coli* (EHEC) O157:H7 Sakai strain isolated during an outbreak in Sakai City, Japan, in 1996 was studied [30].

**Table 1:** **Comparison of TBARS content of twenty-one *E. coli *belonging to different pathovars and phylogenetic groups during exponential growth in pooled human urine and LB broth**

			**Urine**		**LB broth**		**Urine *vs* LB**
**Strains**	**Phylo-groups**	**Virulence groups**	TBARS*	p**	**TBARS**	***p***	***p***
**ABU 57**	A	ABU	9.0 ± 1.7	*p = 0.835*	7.1 ± 0.7	*p = 1.000*	*p = 0.995*
**ABU83972**	B2	ABU	7.3 ± 1.0		6.4 ± 0.1		*p = 1.000*
**ABU 5**	D	ABU	7.2 ± 1.1	*p = 1.000*	6.3 ± 1.1	*p = 1.000*	*p = 1.000*
**IH11128**	B2	UPEC	6.7 ± 2.0	*p = 1.000*	6.1 ± 0.4	*p = 1.000*	*p = 1.000*
**IAI 74**	B2	UPEC	6.6 ± 0.9	*p = 1.000*	6.1 ± 0.7	*p = 1.000*	*p = 1.000*
**ABU 63**	B2	ABU	6.6 ± 1.2	*p = 1.000*	5.8 ± 0.9	*p = 1.000*	*p = 1.000*
**IAI 39**	D	UPEC	6.6 ± 1.5	*p = 1.000*	7.1 ± 0.7	*p = 1.000*	*p = 1.000*
**CFT073**	B2	UPEC	6.3 ± 0.9	*p = 0.999*	5.9 ± 0.7	*p = 1.000*	*p = 1.000*
**ABU 27**	B2	ABU	5.6 ± 0.9	*p = 0.905*	4.9 ± 0.5	*p = 0.976*	*p = 1.000*
**ABU 64**	B2	ABU	5.6 ± 0.4	*p = 0.866*	6.8 ± 0.2	*p = 1.000*	*p = 1.000*
**UMN 026**	D	UPEC	5.2 ± 1.1	*p = 0.458*	6.2 ± 0.6	*p = 1.000*	*p = 1.000*
**ED1a**	B2	commensal	5.2 ± 1.1	*p = 0.542*	4.9 ± 0.2	*p = 0.979*	*p = 1.000*
**536**	B2	UPEC	5.1 ± 1.0	*p = 0.458*	6.3 ± 1.7	*p = 1.000*	*p = 1.000*
**J96**	B2	UPEC	5.0 ± 1.1	*p = 0.653*	5.3 ± 1.4	*p = 0.998*	*p = 1.000*
**ABU 20**	B2	ABU	4.9 ± 0.9	*p = 0.307*	4.3 ± 1.2	*p = 0.743*	*p = 1.000*
**IAI1**	B1	commensal	4.3 ± 0.7	*p = 0.075*	4.4 ± 0.3	*p = 0.789*	*p = 1.000*
**Sakai**	E	EHEC	3.9 ± 0.4	*p = 0.016*	4.6 ± 0.7	*p = 0.900*	*p = 1.000*
**UTI 89**	B2	UPEC	3.8 ± 0.6	*p = 0.015*	5.1 ± 0.1	*p = 0.996*	*p = 1.000*
**ABU 38**	B1	ABU	3.8 ± 0.8	*p = 0.012*	4.5 ± 0.1	*p = 0.838*	*p = 1.000*
**ABU 62**	B1	ABU	3.5 ± 0.9	*p = 0.005*	6.1 ± 0.8	*p = 1.000*	*p = 0.560*
**MG1655**	A	K-12 laboratory strain	2.6 ± 0.5	*p = 0.0001*	6.3 ± 1.4	*p = 1.000*	*p = 0.546*

* TBARS values are expressed in micromoles per 10^11^ cells. The data shown are means (mean ± SD) of three independent experiments in different batches of urine and LB broth. Differences between means were evaluated for statistical significance using the Tukey's HSD (Honestly Significant Difference) test.

**p values of < 0.05 were considered significant. compared to ABU83972 under the same conditions.

### Growth conditions, dissolved oxygen saturation and cell extracts

The study was performed in Luria Bertani (LB) broth, a nutritionally rich medium and in human urine. Three independent batches of urine from healthy volunteers were used. Midstream urine was collected over 24 hours from healthy male volunteers, with no history of UTI or antibiotic use in the last two months. The urine was pooled, centrifuged, filtered (filter size 0.22μm) and stored at −20°C. The growth experiments were assayed using 96 well plates and OD_600_ was measured with a Tecan Infinite M200 plate reader. For the preparation of cell extracts, aliquots of 40 ml of pooled urine or LB broth in 50 ml vials were inoculated with the appropriate volume of the overnight culture to obtain 2 x 10^5^ bacteria/ml. Each experiment was performed in triplicate and repeated in 3 different batches of urine or LB broth. Cells were grown at 37°C under microaerobic conditions (1% O_2_). Dissolved oxygen saturation was measured by luminescence with a measure probe (Hach Lange GmbH) in the different media during the exponential growth phase. The measure was repeated at least four times. Cultures were sampled in mid-exponential growth phase and 30 min after the beginning of stationary phase. Aliquots of 40 ml of culture were centrifuged at 4500 rpm at +4°C for 15 min. The bacteria were washed twice with 0.9% NaCl, pelleted and stored at −20°C until used. The cells resuspended in appropriate sonicating buffers (see below) were disrupted by sonication on ice for 3 min (30 s disrupt with 30 s rest) with an ultrasonic disrupter (Sonics & Materials Inc.).

### Antioxidant enzyme and glutathione assays

The pellets were sonicated in phosphate buffer, pH 7.8. All assays, except catalase activity, were performed on a Roche Diagnostics/Hitachi 912. Catalase activity was determined using the Catalase Assay kit (Sigma). The Cu-SOD activity, which corresponds to the periplasmic SOD, was assayed using the SOD assay kit (Randox laboratories) based on the method of Mc Cord and Fridovich [31]. The cytosolic SOD activity, which is effected by the Mn- and the Fe-SODs, was calculated as the difference between the total SOD activity measured at pH 7.8 and the Cu-SOD activity measured at pH 10.2. The glutathione oxidoreductase was assayed by the method of Bleuter [32]. Oxidized glutathione (GSSG) was added and the disappearance of NADPH was monitored at a wavelength of 340 nm. The assay of glucose-6-phosphate-dehydrogenase (G6PDH) was based on Bleuter’s method [33], where glucose-6-phosphate was added and the reduction of NADP to NADPH was monitored at a wavelength of 340 nm. The γ-glutamylcysteine synthetase (GshA) and the glutathione synthetase (GshB) were assayed as described previously [34]. Briefly, ADP generated by both enzymes in the presence of their substrates was determined using a coupled assay with pyruvate kinase, and lactate dehydrogenase. Oxidized and reduced glutathione concentrations were assayed by high-performance liquid chromatography (HPLC) equipped with a colorimetric detection system, using *N-*acetyl cysteine as an internal control [35]. Each experiment was performed in triplicate and repeated in 3 different batches of urine. The activities of the enzymes and the glutathione content in each sample were normalized with total proteins assayed by the method of Bradford [36].

### Measurement of thiobarbituric acid reactive substances (TBARS)

Lipid peroxidation was estimated as TBARS content. TBARS were measured by the standard procedure using the protocol according to the manufacturer’s instructions (OxiSelectTM TBARS Assay kit, Cells Biolabs). When cells were either in exponential growth phase or in stationary phase, OD_600_ of the cultures and TBARS concentrations were determined. The pellets were sonicated in PBS buffer containing 1% Triton X-100 and 0.05% antioxidant butylated hydroxytoluene to prevent further oxidation of lipid. Each experiment was performed in duplicate and repeated in 3 different batches of human urine and LB broth.

### Statistical analysis

Differences between means of at least 3 to 9 experiments were evaluated for statistical significance using the Tukey's HSD (Honestly Significant Difference) test. Non-parametric data were analysed using a Mann–Whitney U-test. *P* values of < 0.05 were considered significant. Data are presented as mean ± standard deviation or as box-plots based on medians and quartiles.

## Results

### Growth in human urine is limiting

**Figure 2:**
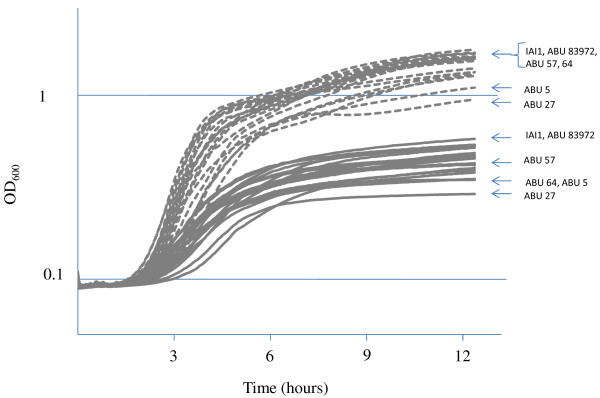
**Growth of twenty-one *E. coli *belonging to different pathovars and phylogenetic groups.** Growth in LB broth (dashed line) and in pooled human urine (complete line). The plotted values are means of 3 independent experiments. OD_600_, optical density at 600 nm. Strains with exponential phase in urine significantly different are specifically labeled.

The growth capacity of twenty-one *E. coli* strains (8 UPEC, 1 EHEC, 9 ABU, 3 commensal strains) was studied (Figure 2). As expected, growth in pooled human urine was significantly less than in LB medium, for all strains and supplementation of urine with casaminoacids improved growth (data not shown). Unlike LB broth, urine limits cell growth. Moreover, in LB broth as in urine, it was found that all strains produced similar growth curves. Only both strains ABU 83972 and IAI1 grew slightly faster than four ABU strains (57, 64, 27 and 5) during the exponential phase in urine (*p* < 0.0001). Surprisingly, the growth capacity of ABU in the urine is not better than that of UPEC and commensal strains.

### The TBARS content differs between strains grown in urine

The content of TBARS, corresponding to the accumulation of membrane lipid peroxidation products was measured during exponential growth in both culture media, pooled human urine and LB broth (Table 1). The levels of damage products accumulated have been used to assess oxidative stress induced by intracellular ROS [16,37]. In all cases, *p* values were versus ABU 83972 strain. No significant difference was observed in TBARS content of twenty-one strains grown in LB broth while differences occurred during growth in urine. Similar amounts of TBARS were produced by ABU 83872 and fourteen other strains. These amounts were significantly higher than those produced by five other *E. coli* strains (Sakai, UTI89, MG1655 and ABU 38 and 62). IAI1 with a *p* value at 0.075 was at an intermediate position. These data show that during exponential growth in urine, the intracellular ROS level differs between strains. Furthermore, the ROS level is not linked to the phylogenetic groups. Strains belonging to different phylogenetic groups were found in both groups with high and low TBARS content, respectively. In the same way, no clear delineation between virulence categories was apparent (Table 1). Interestingly, it also appeared that strains which grew slightly faster or slower in urine e.g. ABU strains 83972 and 64 respectively, exhibited similar levels of TBARS.

### ABU 83972 strain more effectively controls the level of TBARS in urine

Changes in ROS levels produced in the exponential and stationary growth in both pooled human urine and LB broth were studied using a representative panel of strains [three UPEC strains (CFT073, UTI89, 536), all belonging to the phylogenetic B2 group, three commensal strains (ED1a, IAI1, MG1655) belonging to various phylogenetic groups, the ABU 83972 from phylogenetic group B2 and Sakai from phylogenetic group E] (Table 2). Due to the sampling procedure, data obtained were subject to a new analysis of variance. The statistical analysis performed on a limited number of strains showed results quite similar to the first analysis. Similar amounts of TBARS were produced by ABU 83972 and CFT073 during exponential growth in urine. These amounts were significantly higher than those produced by the four strains IAI1, Sakai, UTI89 and MG1655. ED1a and 536 with a *p* value at 0.070 and 0.048 respectively were now at an intermediate position. No significant changes were observed in the stationary phase of growth. As a consequence, similar amounts of TBARS were produced during the two phases of growth except for ABU 83972 in urine. In strain ABU 83972, the level of TBARS was higher in the exponential phase and decreased significantly in the stationary phase showing the ability of strain ABU 83972 to control the endogenous oxidative stress during growth in urine. In contrast, all isolates grown in LB medium exhibited similar levels of ROS regardless of the growth phase.

**Table 2:** **Comparison of TBARS content of eight *E. coli *at both phases (exponential and stationary) of growth in pooled human urine and LB broth**

	**Urine exponential phase**		**Urine stationary phase**		**Urine exponential phase *vs* stationary phase**
**Strains**	TBARS*	p**	**TBARS**	***p***	***p***
**ABU83972**	7.3 ± 1.0		4.4 ± 0.4		*p = 0.014*
CFT073	6.3 ± 0.8	*p = 0.902*	4.7 ± 0.8	*p = 1.000*	*p = 0.450*
ED1a	5.2 ± 1.1	*p = 0.070*	5.2 ± 0.8	*p = 0.927*	*p = 1.000*
536	5.1 ± 1.0	*p = 0.048*	4.1 ± 0.6	*p = 1.000*	*p = 0.993*
IAI1	4.3 ± 0.7	*p = 0.002*	4.6 ± 0.7	*p = 1.000*	*p = 1.000*
Sakai	3.9 ± 0.4	*p = 0.001*	4.2 ± 0.3	*p = 1.000*	*p = 1.000*
UIT89	3.8 ± 0.6	*p = 0.001*	3.9 ± 0.1	*p = 0.997*	*p = 1.000*
MG1655	2.6 ± 0.5	*p < 0.0001*	4.0 ± 1.0	*p = 0.999*	*p = 0.880*
	**LB broth exponential phase**		**LB broth stationary phase**		**LB broth exponential phase vs stationary phase**
**Strains**	**TBARS**	***p***	**TBARS**	***p***	***p***
**ABU83972**	6.4 ± 0.1		8.9 ± 1.6		*p = 0.394*
CFT073	5.9 ± 0.6	*p = 0.993*	6.5 ± 0.4	*p = 0.458*	*p = 1.000*
ED1a	4.9 ± 0.2	*p = 0.492*	6.8 ± 1.2	*p = 0.581*	*p = 0.763*
536	6.3 ± 1.7	*p = 1.000*	5.4 ± 1.9	*p = 0.135*	*p = 0.998*
IAI1	4.4 ± 0.3	*p = 0.219*	6.8 ± 0.1	*p = 0.571*	*p = 0.465*
Sakai	4.6 ± 0.7	*p = 0.316*	6.7 ± 1.1	*p = 0.543*	*p = 0.635*
UIT89	5.1 ± 0.1	*p = 0.656*	7.4 ± 0.4	*p = 0.844*	*p = 0.540*
MG1655	5.5 ± 0.1	*p = 0.907*	7.0 ± 0.1	*p = 0.680*	*p = 0.942*

* TBARS values are expressed in micromoles per 10^11^ cells. The data shown are means (mean ± SD) of three independent experiments in different batches of urine and LB broth. Differences between means were evaluated for statistical significance using the Tukey's HSD (Honestly Significant Difference) test.

**p values of < 0.05 were considered significant. compared to ABU83972 under the same conditions.

The behavior of the commensal strains and UPEC in urine was also compared. As expected, no significant difference was observed in the amount of TBARS produced (data not shown). *E. coli * is a diverse species, both in terms of gene content and sequence divergence [24,38], so we then analysed strains from the phylogenetic B2 group only, which includes both commensal and pathogenic strains. No difference was observed between the UPEC and the ED1a intestinal commensal strains (*p* = 0.968). However, clear differences were demonstrated in urine between the three UPEC strains selected (5.19 ± 1.31) and the ABU strain 83972 (7.26 ± 1.03) with a *p* value = 0.009.

### ABU strain 83972 has better antioxidant defense capacity than UPEC strains

**Figure 3:**
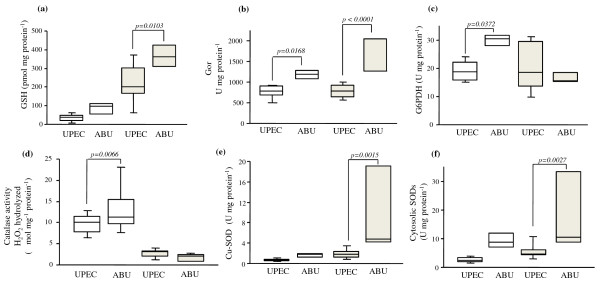
**Comparison of antioxidant defense mechanisms between UPEC (CFT073, UTI 89 and 536) and ABU 83972 strains at both phases of growth.** (**a**) Content of glutathione (GSH), (**b**) Glutathione oxidoreductase (Gor) activity, (**c**) Activity of glucose 6 phosphate deshydrogenase (G6PDH), (**d**) Catalase activity, (**e**) Activity of superoxide dismutase activity cooper-dependent (Cu-SOD), (**f**) Activity of cytosolic superoxide dismutases (cytosolic SODs) (Mn-dependent and Fe-dependent). White square: mid-logarithmic phase; grey square: stationary phase.

The non-enzymatic and enzymatic components involved in antioxidant defense systems (Figure 1b) were studied during growth in pooled human urine in a subset of four B2 UPEC and ABU strains selected from the previous panel (CFT073, UTI89, 536 and ABU 83972) (Additional file 1: Table S1 and Additional file 2: Table S2). To increase the statistical power of our analysis, antioxidant defense mechanisms of the three UPEC were compared with those of ABU 83972. The results are presented Figure 3. We also compared antioxidant defense systems between ABU 83972 and CFT073 alone. Similar results to those obtained for ABU 83972 and the three UPEC were obtained, however, the *p* values were less significant (between 0.03 and 0.15) (data not shown).

#### Glutathione system

The *E. coli* redox buffer in the cytoplasm is mostly composed of the tripeptide glutathione. The intracellular concentration is approximately 5 mM, and it is kept almost completely reduced (GSH). Glutathione oxidoreductase (Gor) reduces glutathione disulphide (GSSG), which is formed upon oxidation, at the expense of NADPH [14].

In urine, GSH content was low in the mid-exponential phase and then increased in all strains (Figure 3a, Additional file 1: Table S1 and Additional file 2: Table S2). However, GSH content was significantly higher for ABU 83972 than for the UPEC in the stationary phase. No significant difference was observed in enzyme-synthesised GSH [γ-glutamylcysteine synthetase (GshA) and glutathione synthetase (GshB)] or GSSG content between UPEC and strain ABU 83972 or between growth phases (Additional file 1: Table S1 and Additional file 2: Table S2). Gor activity was significantly higher for strain ABU 83972 than that of UPEC for all measurements and varied significantly between mid-exponential phase and the stationary phase (Figure 3b, Additional file 1: Table S1 and Additional file 2: Table S2).

#### Enzymes responsible for the detoxification of superoxide radicals and hydrogen peroxide

Strain ABU 83972 growth in urine was associated with higher activity of the H_2_O_2_ detoxification system. Catalase activity represents the peroxidase activity of several enzymes (Figure 1b), such as hydroperoxydase I (HPI), hydroperoxydase II (HPII) and the alkyl hydroperoxydase (AhpC) [39,40]. Catalase activity of strain ABU 83972 was significantly higher in mid-exponential phase and stayed the same in stationary phase, for both groups (Figure 3d, Additional file 1: Table S1 and Additional file 2: Table S2). Enzymes responsible for superoxide radical O_2_^.-^ detoxification were induced more during growth and were also more active in this strain. All superoxide dismutases, periplasmic and cytosolic activity increased significantly during growth, becoming significantly greater in the stationary phase for strain ABU 83972 only (Figure 3e3f). Moreover, glucose-6 phosphate dehydrogenase (G6PDH) activity of strain ABU 83972 was significantly greater in the mid-exponential phase, and decreased to levels similar to those of UPEC in the stationary phase (Figure 3c). This more active G6PDH could contribute to the synthesis of antioxydants (NADPH, GSH).

As shown above, ABU 83972 growth in urine was related to a significantly higher level of TBARS in the mid-exponential phase. The high level of antioxidant defenses of strain ABU 83972 resulted in a decrease of TBARS, so there was no difference in the levels of TBARS in the stationary phase between ABU strain 83972 and CFT073 or three UPEC.

## Discussion

Our studies demonstrate that growth in urine may be associated with endogenous oxidative stress. It is well known that urine supports bacterial growth. Several studies have shown that UPEC strains grow well in human urine, whereas faecal isolates tended to grow more poorly [19,41]. Other studies have also reported that ABU isolates grow faster than UPEC strains [11]. However, Alteri and Mobley have recently shown that growth in urine is not restricted to UPEC bacteria or ABU strains. Commensal and enteropathogen *E. coli* strains produced growth curves indistinguishable from those of UPEC [42]. In agreement with their work, we found that the ability to grow of twenty-one *E. coli* belonging to different pathovars and phylogenetic groups was nearly similar in pooled human urine. The slow growth of a few isolates could reflect the RpoS polymorphism in *E. coli* population [43,44].

Cultures were grown in microaerophilic conditions (see Methods), where oxygen levels are similar to those in the human urinary tract [18,45]. This reduction in oxygen content leads to a redistribution of metabolic fluxes between fermentation and respiration [46]. Such a shift may decrease the respiratory chain-mediated generation of ROS. Moreover, in our culture conditions, autoxidizable enzymes such as L-aspartate oxidase and fumarate reductase should not contribute significantly to the formation of H_2_O_2._ However, metabolic reactions that generate nearly two-thirds of H_2_O_2_ are not yet identified [47]. Therefore, we can expect that changes in metabolic fluxes generate different ROS levels. Analysis of metabolic capabilities in a collection of 153 *E. coli * natural isolates [48] and of gene expression in strains ABU 83972 and CFT073 grown exponentially in urine [49] revealed significant differences in their metabolic capacities. These metabolic changes could therefore generate different ROS levels in our isolates.

Urine is a complex growth medium and *E. coli * must adapt to stress imposed by this tough environment. The high osmolality, high urea concentration, low pH and the limitation of certain components could provoke an oxidative response. To protect from these highly reactive intermediates, cells possess a defense system consisting of both enzymatic and non- enzymatic antioxidants that scavenge them. Nevertheless, under several situations, the rate of generation of ROS exceeds that of their removal and oxidative stress occurs. The levels of damage products accumulated (estimated as TBARS concentration) mirror the intensity of oxidative stress. Our results demonstrate that *E. coli* strains can respond very differently to stress imposed by urine. TBARS measurements revealed that many *E. coli* are exposed to ROS during exponential growth in urine. Surprisingly, this is the case of ABU strain 83972 that is very well adapted to growth in the urinary tract [11]. In contrast, two other ABU strains 38 and 62, as UTI89, Sakai and MG1655 showed a lower oxidative damage to lipid. No clear correlation between ROS level and the phylogroups or pathogenic group was apparent.

ABU isolates form a heterogenous group. Individual ABU strains display many differences between them in their genome contents and in virulence-associated genes such as LPS, microcin, aerobactin, and mobility [11,27]. Interestingly, our study shows that the two ABU strains (38 and 62) belonging to the group B1, differ from other by the low ROS production in urine.

The commensal-like ABU 83972 strain and the pathogenic strain CFT073 are very closely related and belong to the same B2 subgroup II [25], or to the same sequence type 73 clonal group [4]. These both strains are genetically very similar and differ only by a few hundred genes [50]. However, strain ABU 83972 is able to outcompete CFT073 strain in urine [51]. The results presented herein indicate that both strains undergo an oxidative stress during the exponential growth. Nonetheless, ABU 83972 strain displays more active antioxidant defenses which led to a significant decrease in ROS level in stationary phase. Our results agree with the gene expression profiling in strains ABU 83972 and CFT073 in urine, which showed that *sodA*, encoding superoxide dismutase and *ahpC*, encoding hydroperoxide reductase are significantly up-regulated [49,52]. Interestingly the highest expression values were obtained in ABU 83972 strain [49]. To further explore the oxidative response, other studies will be performed to examine the contribution of each factor involved in this response and the importance of metabolic changes in these isolates.

The UPEC strains CFT073 (urosepsis/pyelonephritis isolate), 536 (pyelonephritis, B2 subgroup III) and UTI89 (cystitis, B2 subgroup IX) [25] are very well adapted for growth in the human urinary tract and present similar antioxidant defense systems. However, a clear distinction can be drawn between them. Strains CFT073 and 536 behave similarly with respect to ROS formation in exponential phase in contrast to UTI89 (*p* = 0.016). The metabolic fluxes could be distributed differently in UTI89, which may decrease the endogenous production of ROS.

The more efficient antioxidant metabolism related to greater exposure to endogenous oxidative stress may be responsible for the difference in lifestyle between ABU 83972 and CFT073 strains. ABU 83972 strain exploits urine more efficiently than UPEC strains [11]. Previous study has shown that a more active antioxidant defense system increases the capacity to colonize the bladder [53]. Thus, a high level of antioxidant defenses associated to fast growth in the urine (this work), low abundance of fimbriae, and possible biofilm formation [54] could explain why ABU83972 strain is able to establish a long-term bacteriuria. Additionally, ROS are implicated in DNA mutagenesis which may be adaptive as reported in biofilm for antibiotic-resistance [55], or more generally, during starvation [56]. The high levels of ROS in ABU strain 83972 may explain the genetic alterations described [27].

## Conclusions

We showed that growth in human urine of many *E. coli* strains belonging to different phylogenetic groups and pathovars was associated with an endogenous oxidative stress. The growth of ABU strain 83972 was associated with a high level of ROS and more active antioxidant defenses. The increased level of ROS may be responsible for adaptive mutations. A more active antioxidant defense system could increase the capacity to colonize the bladder.

## Authors’ contributions

CA, JG, CM, MC performed the research. CA, OH, MC, OB analysed the data. DB, JD, UD, ED participed to the coordination of the study. OB wrote the paper. All authors read and approved the final manuscript.

## Supplementary Material

Additional file 1: Table S1 Comparison of the antioxidant defense systems in three UPEC (CFT073, UTI89, 536) and ABU 83972 strains during the mid-logarithmic growth phase in urine. Click here for file

Additional file 2: Table S2 Comparison of the antioxidant defense systems of three UPEC (CFT073, UTI89, 536) and ABU 83972 strains during the stationary growth phase in urine. Click here for file
