# Supplementary material for: Variation in endogenous oxidative stress in Escherichia coli natural isolates during growth in urine
Source: BMC Microbiol. 2012 Jun 22;12:120. doi: 10.1186/1471-2180-12-120 (PMC3479029; doi:10.1186/1471-2180-12-120)
Supplement: Additional file 1: Table S1 — Comparison of the antioxidant defense systems in three UPEC (CFT073, UTI89, 536) and ABU 83972 strains during the mid-logarithmic growth phase in urine. [file 1471-2180-12-120-S1.doc]

**Table S1:** **Comparison of the antioxidant defense systems in three UPEC (CFT073, UTI89, 536) and ABU 83972 strains during the mid-logarithmic growth phase in urine**

| Variable | UPEC  (mean ± SD) | ABU  (mean ± SD) | *p* |
| --- | --- | --- | --- |
| GSH (pmol. mg protein^-1^) | 35.5 ± 20.2 | 84.3 ± 39.8 | *0.380* |
| GSSG (pmol. mg protein^-1^) | 2.8 ± 3.8 | 2.7 ± 3.5 | *0.980* |
| GshA (mU. mg protein^-1^) | 0.9 ± 0.1 | 1.0 ± 0.2 | *0.412* |
| GshB (mU. mg protein^-1^) | 1.1 ± 0.2 | 1.2 ± 0.2 | *0.482* |
| Gor (mU. mg protein^-1^) | 0.8 ± 0.1 | 1.2 ± 0.1 | *0.017* |
| G6PDH (U. mg protein^-1^) | 19.1 ± 3.8 | 29.8 ± 2.4 | *0.037* |
| Cu-SOD (U. mg protein^-1^) | 0.7 ± 0.3 | 1.6 ± 0.6 | *0.720* |
| Cytosolic SODs (U. mg protein^-1^) | 2.6 ± 0.9 | 9.2 ± 3.2 | *0.014* |
| Catalase activity (mmol min^-1^ mg protein^-1^) | 10.1 ± 3.2 | 13.4 ± 5.8 | *0.006* |

GshA = γ-glutamylcysteine synthetase, GshB = glutathione synthetase, Gor =glutathione oxidoreductase, Cu-SOD = copper dependant superoxide dismutase, Cytosolic SODs = manganese dependent superoxide dismutase and fer dependent superoxide dismutase.
