# Supplementary material for: Variation in endogenous oxidative stress in Escherichia coli natural isolates during growth in urine
Source: BMC Microbiol. 2012 Jun 22;12:120. doi: 10.1186/1471-2180-12-120 (PMC3479029; doi:10.1186/1471-2180-12-120)
Supplement: Additional file 2: Table S2 — Comparison of the antioxidant defense systems of three UPEC (CFT073, UTI89, 536) and ABU 83972 strains during the stationary growth phase in urine. [file 1471-2180-12-120-S2.doc]

**Table S2: Comparison of the antioxidant defense systems of** three UPEC (CFT073, UTI89, 536) and ABU 83972 strains during the stationary growth phase in urine

| Variable | UPEC  (mean ± SD) | ABU  (mean ± SD) | *p* |
| --- | --- | --- | --- |
| GSH (pmol. mg protein^-1^) | 215 ± 81 | 365 ± 76.3 | *0.010* |
| GSSG (pmol. mg protein^-1^) | 0.7 ± 0.4 | 0.4 ± 0.3 | *0.846* |
| GshA (mU. mg protein^-1^) | 0.8 ± 0.2 | 0.9 ± 0.1 | *0.825* |
| GshB (mU. mg protein^-1^) | 1.0 ± 0.3 | 1.1 ± 0.2 | *0.652* |
| Gor (mU. mg protein^-1^) | 0.8 ± 0.1 | 1.6 ± 0.5 | *0.0001* |
| G6PDH (U. mg protein^-1^) | 20 ± 8 | 17 ± 2 | *0.440* |
| Cu-SOD (U. mg protein^-1^) | 1.9 ± 1 | 11 ± 11 | *0.001* |
| Cytosolic SODs (U. mg protein^-1^) | 5.7 ± 3 | 19 ± 18 | *0.003* |
| Catalase activity (mmol. min^-1^ mg protein^-1^) | 2.9 ± 1 | 1.7 ± 1.1 | *0.480* |

GshA = γ-glutamylcysteine synthetase, GshB = glutathione synthetase, Gor =glutathione oxidoreductase, Cu-SOD = copper dependant superoxide dismutase, Cytosolic SODs = manganese dependent superoxide dismutase and fer dependent superoxide dismutase.
